# A report from the Irish women in cardiology survey, exploring Europe’s largest gender gap in cardiology

**DOI:** 10.1093/ehjopen/oeac033

**Published:** 2022-05-10

**Authors:** Bethany Wong, Alice Brennan, Stephanie James, Lisa Brandon, Deepti Ranganathan, Barbra Dalton, Ken McDonald, Deirdre Ward

**Affiliations:** School of Medicine, University College Dublin, Dublin 4, Ireland; Department of Cardiology, Golden Jubilee Hospital, Glasgow, UK; Department of Cardiovascular Imaging, Beacon Hospital, Dublin, Ireland; Department of Cardiology, St James Hospital, Dublin, Ireland; Department of Cardiology, Mater Misercordiae Hospital, Dublin, Ireland; Irish Cardiac Society, 17-19 Lower Rathmines Road, Dublin, Ireland; School of Medicine, University College Dublin, Dublin 4, Ireland; Department of Cardiology, Tallaght Hospital, Dublin, Ireland

**Keywords:** Women, Sexism, Discrimination, Flexible-training, Mentorship, Cardiology

## Abstract

**Aims:**

In Ireland, 8% of public cardiology consultants are female; this is the lowest proportion in Europe. We sought to understand perceptions amongst Irish trainees and consultants regarding aspects of working in cardiology in order to identify areas that can be targeted to improve gender equality.

**Methods and Results:**

In September 2021, the Irish Cardiac Society distributed a questionnaire to trainees and consultants in the Republic and Northern Ireland. Ethical approval was obtained from the University College Dublin, Ireland. There were 94 respondents (50% male, 50% consultants) which equates to ∼30% of all trainees and consultants in all Ireland. Although females were more likely to be single, overall, they had additional child-care responsibilities compared with male counterparts. Despite 53% of the respondents preferring to work less than full time, 64% reported a perceived lack of support from their departments. Males were significantly more likely to go into procedural/high radiation sub-specialities. Bullying was reported by 53% of females. Almost 80% of females experienced sexism and 30% reported being overlooked for professional advancement based on their sex. Females also rated their career prospects lower than males. Key challenges for women were: sexism, maternity leave/child-care responsibilities, cardiology as a ‘boys club’ and lack of flexible training. There was interest from both males and females in a mentorship programme and support for women in leadership positions.

**Conclusion:**

Discrimination including sexism, bullying, and equal opportunity for professional advancement are key aspects that need to be addressed to improve gender balance in cardiology within Ireland and Northern Ireland.

## Introduction

In the Republic of Ireland (ROI), 8% of public cardiology consultants are female; the lowest proportion in Europe, despite more females entering the medical workforce. The Medical Workforce Report 2020–21^1^ stated that cardiology had the lowest female consultant ratio of any medical sub-speciality.^1^ Although, Northern Ireland (NI) is part of the UK, NI and ROI share an ‘all island’ professional society called the Irish Cardiac Society (ICS). Although there are currently no data as to the female consultant ratios in NI, it is likely similar to both the UK (13% female consultants^2^) and ROI.

Several publications examining the gender gap in cardiology globally^2–5^ have aimed to find solutions to improve gender discrepancy. Although Ireland ranks in the top 10 most gender-equal countries in the world, with almost gender parity on educational attainment (99.8%) and Health and Survival (96.4%),^6^ this does not translate into cardiology. We sought to understand the perceptions of Irish trainees and consultants on aspects of working in cardiology to identify areas that can target this disparity.

## Methods

University College Dublin research ethics committee approved this study. A questionnaire was created and adapted from previous studies assessing the reasons for gender discrepancies in cardiology.^2–4,7^ Irish Cardiac Society, distributed the survey to all cardiology trainees and consultants through its mailing list.

Descriptive and frequency analyses were used for demographic data. Comparisons between groups were conducted using independent samples *t*-tests or Mann–Whitney *U* tests. *χ*^2^ tests were used to analyse gender response differences. Phi coefficient was used for assessing the effect size of *χ*^2^ associations, with an effect value of 0.1 = small effect, 0.3 = moderate effect, 0.5 = large effect size. Two-sided *P*-values <0.05 were considered statistically significant. Themes for free text box answers were collated. Only one theme per participant response was allocated to ensure equal representation.

## Results

There were 94 respondents with a response rate of 30%. *Table 1* demonstrates a comparison of baseline characteristics between males and females. Females made up 47 (50%) of respondents. A total of 54% were married, however, females were more likely to be single compared with their male counterparts (36% vs. 17%, *P* < 0.05). Females also reported higher levels of childcare responsibilities, with 19% providing >70% of the childcare duties, vs. 5% of males reporting to provide >70% of the childcare duties (*P* < 0.05, phi = 0.42).

**Table 1:** Baseline comparison of male and female characteristics

		Female		Male		Total		*P-*value
		*n*	%	*n*	%	*n*	%	
Baseline characteristics		47	50	47	50	94	100	
Ethnicity	Non-white	8		8		16	17	
	White	39		39		78	83	
Trainee or consultant?	Consultant	21	45	22	47	43	46	
	Non-consultant	26	55	25	53	51	54	
What is your current marital status?	Divorced	1	2	1	2	2	2	
	Married	20	43	31	66	51	54	
	Not married, living with partner	7	15	7	15	14	15	
	Single	17	36	8	17	25	27	*P* <0.05
Do you have any children (include any children whether biological or adopted)?	No	28	60	22	47	50	53	
	Yes	19	40	25	53	44	47	
If you do have children, how many?	0	28		22		50	53	
	1	6		6		12	13	
	2	4		5		9	10	
	3	4		8		12	13	
	4	3		5		8	9	
	5	0		1		1	1	
Proportion of childcare	≤30%	2	5	11	26	13	31	*P* <0.05
	31–49%	1	2	7	17	8	19	
	50%	3	7	3	7	6	14	
	51–69%	4	10	1	2	5	12	
	≥70%	8	19	2	5	10	24	*P* <0.05
Working full or part-time	Full-time	46		47		93	99	
	Part-time	1		0		1	1	
Sub-specialisation	High procedural (intervention/EP)	13	14	26	28	39	41.5	*P* <0.05
	Low procedural (HF/imaging)	20	21	11	12	31	33	*P* <0.05
	Other (congenital/academic/preventive/ICC)	14	15	10	11	24	25.5	

Despite only one person (1%) reporting working less than full time (LTFT), the majority (53%) of the respondents said they would consider working LTFT, given the opportunity. The main reasons were more time with family, better work–life balance, and burnout. However, almost two-thirds (64%) of respondents felt their departments would not accommodate LTFT cardiologists.

A variety of sub-specialty fields within cardiology were represented including interventional cardiology (30%), imaging (20%), heart failure (13%), electrophysiology (12%), and general cardiology (16%). Of note, males were twice as likely (28% vs. 14%, *P*-value <0.05, phi = 0.29) to choose specialities involving higher radiation exposure, such as intervention or electrophysiology.

Forty-eight per cent of respondents reported having experienced bullying, regardless of gender (females 53%, males 43%) or seniority. Consultants accounted for 60% of bullies. Only 1 in 2 respondents reported bullying to seniors (53%), and 46%, felt a lack of reporting system.

**Figure 1:**
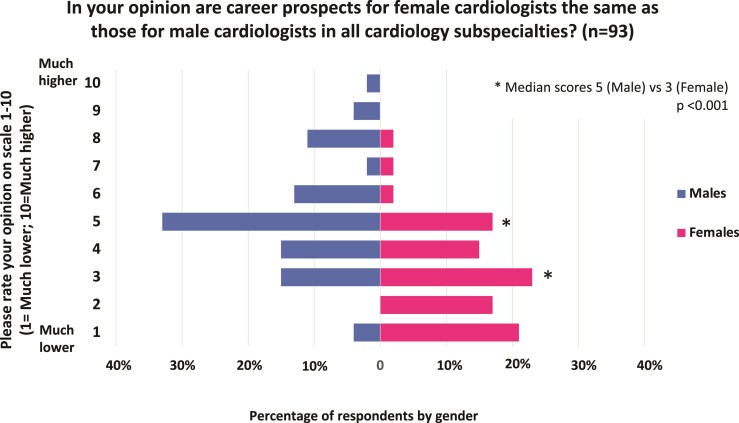
Comparison of perception of career prospects for female cardiologists between males and females. The figure demonstrates a scale from 1 to 10 of respondents answer to the question ‘In your opinion are career prospects for female cardiologists the same as those for male cardiologists in all cardiology sub-specialties?’ Where 1 is much lower and 10 is much higher. The lower the score, the lower the perceived career prospects for females compared with males. Females reported a median score of 3, which is a significantly lower than the median score of male respondents. Males reported (with a median score of 5), that career prospects for females are the same as males.

A total of 79% of females reported experiencing sexism, compared with 15% of males (*P* < 0.001, phi = 0.65). There was a significant difference (*P* = 0.001, phi = 0.40) in females (30%) compared with males (2%) reporting, missed opportunities for professional advancement based on their gender. Most females (85%) felt that training in cardiology was harder for female trainees, and this view was shared by 53% of male respondents. *Table 2* demonstrates themes and example responses to the question: ‘Why is it more difficult to train in cardiology as a female?’. Each respondent reported their top reason. Themes included: sexism (19%), maternity difficulties (19%), childcare commitments (19%), cardiology being perceived as a male-dominated speciality (14%), and a lack of work flexibility (13%) as key reasons. *Figure 1* demonstrates that females report that their career prospects were significantly lower than males, whereas males reported that career prospects were the same.

**Table 2:** Ranked Responses to ‘Why do you think it is more difficult for females to train in cardiology than males?

Theme	No. of responses in theme	Percentage responses (%)	Example responses
Sexism	12	19	Cultural and societal gender bias is still a significant problem.
			Predominantly male consultants demonstrate preference to working with male trainees.
			Female mistakes, decisions questioned and judged to much higher degree than their male counterparts mistakes and decisions. Must always excel to be considered acceptable standard—same does not apply for male counterparts.
			Colleagues and patients inherently have more respect for our male counterparts.
Child-care responsibilities	12	19	Child care is also an issue as the hours are long and call is frequent, therefore, difficult to get child care to cover these hours. Also, breast feeding is not feasible when working.
			Lack of adequate supports for childcare, both financial and provision of care.
			That depends if the female trainee has children or not. A female trainee with no children will not find it harder than a male trainee. I think female trainees with children will definitely find it harder to train.
Maternity	12	19	Personal commitments are seen as a hindrance to further career (e.g. maternity leave perceived as ‘unpaid leave’ and expectations to do a research/MD to validate your time ‘off’).
			Time out of training for pregnancy/childbirth/early life care which men do not have.
			If you get pregnant you chose not to go into the cath lab to reduce your radiation exposure and, therefore, your skills in the laboratory and your Logbook will be affected. Also, this affects the on call rota regarding STEMIs.
‘Boys club’	9	14	Machismo still dominates in cardiology despite what might be claimed and I think female peers get a harder time.
			The cardiology consultant community is less open to women—a ‘boys clubs’ which women find harder to access. Many established male cardiologists do not perceive women as ‘one of the lads’.
			A general ‘boys club’ atmosphere when training in cardiology in Ireland. Interventional cardiology is given more credibility than other sub-specialities and this has a very male dominated atmosphere.
Training or work flexibility	8	13	Too fixed a training scheme re relocation, family splitting up, inability to train half time etc.
			Lack of less than full time training opportunities, lack of fellowship possibilities with less than full-time training. All these things do apply to any high profile professional occupation.
			Very, very difficult to go abroad on unpaid fellowships as a mother, both logistically and financially.
			Long hours expected to train, publish, present, travel abroad for training, all not conducive to having children.
Less opportunities	4	6	Bias in access to opportunity/mentoring.
			Female trainees are not given the same learning opportunities, making it harder to upskill in cardiology.
Radiation	4	6	Concern re radiation exposure during pregnancy.
Other	3	5	Training directed in cardiac intervention. Other choices not easily explorable depending which cardiac speciality available in the working hospital.
			Lack of role models.

Overall, 70% of respondents, felt that cardiology would benefit from more female representation and having a mentor through their cardiology training (females = 91%, males = 54%).

## Discussion

Irish female cardiology trainees and consultants report having experienced sexism (79%), bullying (53%), and a perceived lack of career advancement based on gender (30%). There are some similarities to the current study with the British Junior Cardiology Association (BJCA) report.^2,8^ This includes the significant gender difference in those pursuing procedural, high radiation, sub-specialities such as intervention or electrophysiology. In the UK, 9.4% of female trainees^2^ and 48% of female consultants^7^ experienced or witnessed sexism. This is lower than the 79% reported here, and additionally, this all-Ireland survey, asked respondents only if they themselves had experience sexism, and not if it had been witnessed, which would likely lead to a much higher reported rate. Despite rates of bullying in the UK being significantly lower (11% vs. 48%) consultants were the majority of perpetrators in both studies.^8^

In the UK, 9% of cardiology consultants and 6% of trainees work LTFT.^9^ In Ireland, to date there has been only one job share between two trainees since the inception of the cardiology training scheme in 1994 and the number of LTFT consultant cardiologists is <1%. This lack of flexibility in training creates an adverse environment for Irish Cardiologists, especially for women due to childcare responsibilities.

Despite a higher proportion of female cardiology trainees than ever before, a recent study demonstrated that gender parity in cardiology would not be reached in the next 50 years at this current rate.^10^ However, progress in Ireland could be faster given the national gender parity in other domains,^6^ which potentially allows restructuring of available resources and policies from non-medical fields. Interestingly, recent evidence suggests that current national gender parity and its relationship with equality in cardiology is not straightforward, and indeed may have an inverse relationship. Recent work performed by the Pink International Young Academy of Cardiology group demonstrated that in Europe, countries with the most national gender parity had the worst representation of female leaders in cardiology.^5^ Conversely, Russia and Morocco who have the most female cardiology leaders, have the worst parity in gender nationally.

There are some limitations to this study. Firstly, there was participation bias with 50% female response rate, which is a higher representation than their proportion amongst Irish cardiologists. This bias is difficult to address, as the theme of this survey would appeal to women and those who have been affected by discrimination, making them more likely to partake. There was, however, under representation of males and consultants. Despite this, almost all female cardiology trainees and consultants in ROI/NI completed this survey, suggesting rates of sexism, bullying, and perception of lack of career advancement is a true reflection of female Irish cardiologists. Even though this survey had a good response rate of 30%, compared with other gender-based cardiology surveys (response rates 20–23%),^4,7^ it is unlikely to represent the experience of all cardiology trainees or consultants. Finally, another element that was not captured in this survey but has been well documented is salary discrepancies between sexes^3,11^ as well as sexual harassment.^7^

Programme directors in the USA have implemented strategies to promote gender diversity within their programmes.^12^ This includes implicit bias education, prioritizing diversity and equity in developing the match and interview process, and highlighting diversity initiatives in institutions. Some of these strategies could be adopted in Ireland. A fundamental gap highlighted in this survey is the lack of support structure to report discrimination, bullying, and harassment, without the fear of retaliation or stigmatization. This can be challenging, as each hospital human resources department, have different systems for reporting, none of which are anonymised and often do not result in any repercussions to the perpetrator. To target this, changing system-wide policies is needed. These policies should also prioritize systems and facilities to institute family friendly work environments which are already in place in non-medical fields in Ireland. Finally, improving women in leadership roles and mentorship of trainees is critical.

Following on from this survey, Irish Women in Cardiology (WiC) have collaborated with BJCA WiC group and have set up a formalized mentorship programme. Currently, this has enrolled junior doctors, but we aim to extend recruitment to medical schools and subsequently secondary schools to target younger females who have yet to decide on a career. A step in improving equity in leadership positions has already started with a female consultant now sitting on the 10-person selection panel for ROI cardiology specialist training interviews and there is now an equal representation of female council members in ICS for the first time.

In conclusion, this study solidifies themes surrounding why women do not pursue a career in cardiology and presents real-life data on difficulties experienced in day-to-day clinical practice. This includes sexism, bullying, lack of flexible training, maternity, and childcare responsibilities as well as a ‘boys club’ environment that creates a glass ceiling.

## Figures and Tables

**Figure:**
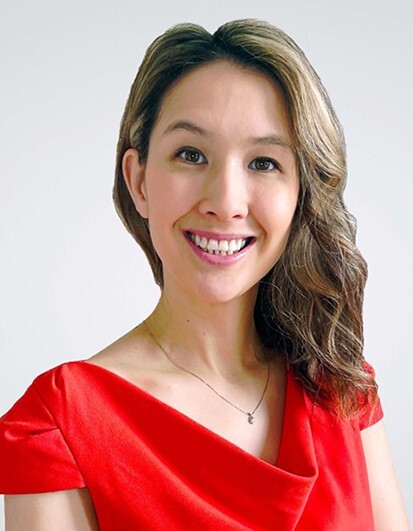


## Data Availability

The data underlying this article will be shared on reasonable request to the corresponding author.

## Lead author biography

Dr Bethany Wong is currently a Cardiology Specialist Registrar, a PhD candidate at University College Dublin and an Irish Clinical Academic Training (ICAT) fellow. She is a founding member of the Irish Women in Cardiology subgroup of the ICS. Through this, she has helped to set up a formalized mentorship programme for junior doctors, as well as fundraise for educational and networking events to improve gender equality in Irish Cardiology.
